# Comparing STRATIFY JCV™ DxSelect™ and IMMUNOWELL™ JCV Tests in RRMS to Assess PML Risk

**DOI:** 10.2174/011570159X372688250226110925

**Published:** 2025-04-07

**Authors:** Aurora Zanghì, Fabiana Marinelli, Paola Sofia Di Filippo, Carlo Avolio, Emanuele D’Amico

**Affiliations:** 1 Department of Medical and Surgical Sciences, University of Foggia, Foggia, Italy;; 2 Neurology Unit, MS Center, F. Spaziani Hospital, ASL Frosinone, Italy

**Keywords:** PML risk, JC virus, IMMUNOWELL™ JCV, STRATIFY JCV™ DxSelect™, natalizumab, antibody test, immunosuppressant drugs

## Abstract

**Background:**

The risk of developing progressive multifocal leukoencephalopathy (PML), a rare but potentially fatal opportunistic infection of the central nervous system caused by the J.C. virus (JCV), remains a primary concern associated with natalizumab therapy in the clinical management of patients diagnosed with multiple sclerosis (MS).

**Materials and Methods:**

This study compared two tests, STRATIFY JCV™ DxSelect™, and IMMUNOWELL™ JCV antibody tests, for assessing the risk of PML in patients diagnosed with relapsing-remitting multiple sclerosis (RRMS) who had received disease-modifying therapy (DMT) with branded natalizumab (Tysabri^®^). The main objective was to determine the comparability of these tests in classifying PML risk. Eligible patients were selected from a database, and all specimens for the STRATIFY JCV™ DxSelect™ and IMMUNOWELL™ JCV antibody tests were collected on the same day. Patients were classified into three risk categories (low, intermediate, or high) based on threshold values for each test.

**Results:**

The analysis showed 85.5% agreement between the two tests for risk classification. Ten discordant cases were identified, mainly between intermediate- and high-risk categories. Compared to STRATIFY JCV™ DxSelect™, IMMUNOWELL™ JCV antibody test tended to categorize more patients as higher risk. No significant association was found between discordance and prior use of immunosuppressant drugs and >24 doses of natalizumab. The agreement between tests, assessed with the weighted Cohen’s Kappa coefficient, was substantial (κ=0.6222).

**Conclusions:**

Compared to the STRATIFY JCV™ DxSelect™, the IMMUNOWELL™ JCV test tends to place more patients in higher risk categories. Further, longitudinal studies are needed to evaluate the clinical impact of these differences in PML risk assessment.

## 1 INTRODUCTION

The expiration of natalizumab's patent (Tysabri^®^) and the subsequent introduction of its biosimilar, Tyruko^®^, has brought new considerations to the forefront of relapsing-remitting multiple sclerosis (RRMS) management, particularly in terms of treatment choice and risk assessment in patients facing disease-modifying therapy (DMT) [1, 2].

Of primary concern during natalizumab therapy in the clinical management of patients diagnosed with RRMS is the drug-associated risk of developing progressive multifocal leukoencephalopathy (PML), a rare but potentially fatal opportunistic infection of the central nervous system caused by the J.C. virus (JCV) [3-5]. PML risk in natalizumab-treated patients has been associated with several factors, including JCV antibody status, prior immunosuppressant use, and duration of natalizumab treatment [6, 7].

While lower-priced biosimilars could make treatment more widely accessible, the choice between the established drug and its biosimilar may pose other implications for PML risk management [1, 8]. This complexity arises from differences in the risk assessment tools that the companies offer and the monitoring protocols associated with each product [9].

Branded natalizumab (Tysabri^®^) has a well-established risk stratification algorithm and monitoring protocol that includes testing with STRATIFY JCV™ DxSelect™ [10]. At the same time, the biosimilar Tyruko^®^ introduces a new testing method, IMMUNOWELL™ JCV, for assessing JCV antibody status [11].

It is important to consider the evolution of the two JCV antibody testing methodologies when comparing them for risk stratification. The STRATIFY JCV™ DxSelect™ methodology has evolved through distinct iterations, with the current DxSelect version representing a second-generation assay [12, 13]. The establishment of its cut-off values has been validated through extensive population-based testing with risk stratification algorithms refined through post-marketing surveillance data establishing standardized index values and risk categories based on quantitative antibody levels, duration of natalizumab exposure, and prior immunosuppressant use [12, 13].

In contrast, the IMMUNOWELL™ JCV antibody assay represents a more recent addition to the diagnostic landscape [14]. While both assays target JCV antibodies, differences in methodological approach and interpretation thresholds necessitate careful attention in clinical practice. Our study focuses on the simultaneous collection of samples for both STRATIFY JCV™ DxSelect™ and IMMUNOWELL™ JCV antibody tests from patients treated with natalizumab (as Tysabri^®^). By analyzing samples collected on the same day with both methodologies, we aimed to determine the comparability of these tests in classifying PML risk during treatment.

## 2 METHODS

### 2.1 Study Design and Population

This was an independent retrospective cohort study. RRMS was diagnosed according to revised McDonald criteria [15]. Patients who were actively followed at two Italian MS centers (Foggia and Frosinone), who were treated with Natalizumab (Tysabri^®^), and who were clinically stable were eligible for enrollment. Clinical data were collected retrospectively by review of the electronic medical record (iMed^©^ software; iMed, Geneva, Switzerland).

### 2.2 Study Outcomes

The main outcome of the study was to determine the comparability of the two tests in classifying PML risk.

### 2.3 Covariates

The dataset contained patient characteristics and treatment histories, including the number of natalizumab administrations, previous immunosuppressive therapy, extended dose regimen, and test results for both STRATIFY JCV™ DxSelect™ and IMMUNOWELL™ JCV antibody tests collected on the same date [16].

Patients were defined as clinically stable if they had no clinical relapses or magnetic resonance imaging activity, such as an increase in the number or volume of brain lesions on T2-weighted sequences or the presence of new contrast-enhancing lesions on T1-weighted gadolinium-enhanced sequences in the previous 12 months. The test thresholds were defined in accordance with the cutoffs established by the respective manufacturers [10, 11]. For STRATIFY JCV^TM^ DxSelect^TM^, a negative result was defined as a value <0.20. For IMMUNOWELLTM JCV, a negative result was defined as a value ≤ 0.20.. Indeterminate results (ranging from ≥ 0.20 to ≤ 0.40 for STRATIFY JCV^TM^ DxSelect^TM^ and from > 0.20 to ≤ 0.50 for IMMUNOWELL™ JCV) required an additional verification test, which provided a final positive or negative result. Specifically, STRATIFY JCV™ DxSelect™ risk categories are defined as: high risk: titer > 1.5, intermediate risk: 0.9 < titer ≤ 1.5; low risk: titer ≤ 0.9 [11]. IMMUNOWELL™ JCV risk categories are defined as high risk: titer > 1.4, intermediate risk: 0.8 < titer ≤ 1.4, and low risk: titer ≤ 0.8 [17].

### 2.4 Statistical Analyses

The analysis involved creating contingency tables to compare the risk classifications provided by the two tests. For each test, the patients were categorized into the three risk classes (low, intermediate, or high) based on their titer values, and the contingency tables were used to calculate the percentage of patients whose risk classification changed between the two tests. Visualization techniques included heatmaps to illustrate risk assessment distributions and radar charts to compare multiple parameters across risk categories.

We conducted a series of analyses to investigate the factors influencing discordance between the tests. First, we calculated the correlation between the STRATIFY JCV™ DxSelect™ titer and the IMMUNOWELL™ JCV titer by applying Pearson’s correlation coefficient. We then examined the association between discordance and the categorical variables of previous immunosuppressive drug use, >24 doses of natalizumab (as Tysabri^®^), and extended dose regimen by using chi-square tests of independence.

Weighted Cohen’s Kappa was used to evaluate the level of agreement between two tests to account for the possibility of agreement occurring by chance. The strength of agreement was interpreted according to Landis and Koch benchmarks [18], where κ-values are categorized as follows: poor (κ<0.00), slight (κ 0.00 to 0.20), fair (κ 0.21 to 0.40), moderate (κ 0.41 to 0.60), substantial (κ 0.61 to 0.80), and almost perfect (κ 0.81 to 1.00). It is particularly useful when the categories are ordinal, as it assigns different weights to disagreements based on their severity.

All statistical tests were conducted with a significance level of α=0.05.

Python 3.11.6 with pandas, matplotlib, and seaborn libraries was used for data analysis and visualization [19].

## 3 RESULTS

A total of 69 patients, mean age of 39±10.3 years, were enrolled (Table **1**). In this cohort, 56 (56/69; 81.2%) tested negative with STRATIFY JCV^TM^ DxSelect^TM^, and 45 (65.2%) tested negative with IMMUNOWELL^TM^ JCV (χ^2^=3.69, *p*=0.0546). The risk classifications (low, intermediate, and high) according to test titers are shown in Table **2**. The level of agreement for risk stratification was 85.5%. Ten discrepancies were recorded in risk levels between the two tests. In detail, the intermediate and high-risk categories showed discordances (*p*<0.05), and the most common type of discordance was between low and intermediate risk (77.8%), followed by low/high classification discordance (22.2%).

**Fig. (1):**
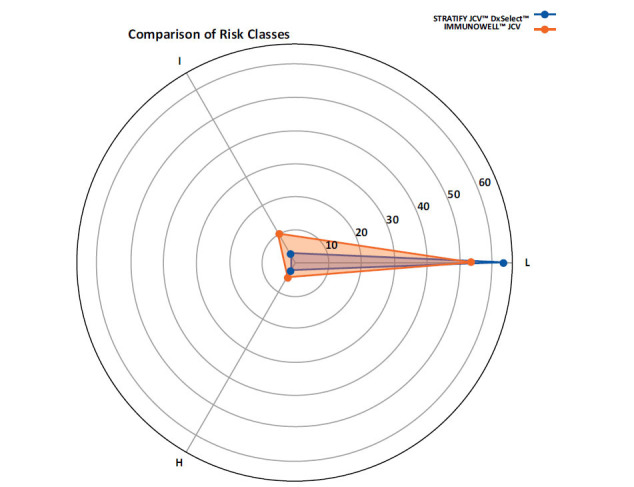
Radar chart for the distribution of risk classes (Low, Intermediate, High) for both STRATIFY JCV™ DxSelect™ and IMMUNOWELL™ JCV tests. Blue lines= STRATIFY JCV™ DxSelect™, orange lines= IMMUNOWELL™ JCV. L=Low; I=Intermediate; H=High.

**Fig. (2):**
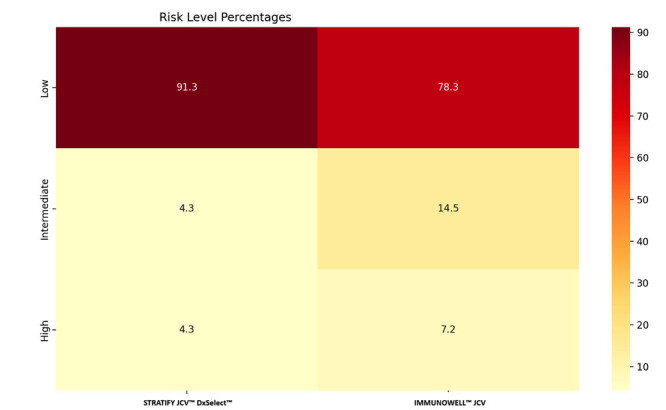
The heatmap displays the percentage distribution of PML risk levels as assessed by STRATIFY JCV™ DxSelect™ and IMMUNOWELL™ JCV tests. The X-axis shows test types, Y-axis indicates risk levels (Low, Intermediate, High). Color intensity corresponds to the percentage of patients in each risk category, with darker colors representing higher percentages. Cell values show the percentage of patients in each category, rounded to one decimal place. n = 69 patients.

The radar chart highlighted the distribution of risk changes, emphasizing the tendency of IMMUNOWELL™ JCV to classify patients into higher risk categories (Fig. **1**). The heatmap (Fig. **2**) confirms these results. STRATIFY JCV™ DxSelect™ test categorized the majority of patients (91.3%) as low risk, with equal distributions (4.3%) in both intermediate- and high-risk categories. In contrast, IMMUNOWELL™ JCV classified 78.3% as low risk while identifying higher proportions in both intermediate- (14.5%) and high-risk (7.2%) categories. This shift in risk categorization between the two methods suggests a more conservative classification approach by IMMUNOWELL™ JCV, particularly evident in the intermediate risk group, where the difference between tests was most pronounced (14.5% *vs.* 4.3%).

The analysis revealed a strong positive correlation (r=0.7495) between the two titers.

Chi-square tests did not show a statistically significant association between discordance and the clinical variables examined (previous DMT use: χ^2^=0.0375, *p*=0.8464; >24 doses of Tysabri^®^: χ^2^=0.0000, p=0.9959; extended dose regimen: χ^2^=0.0507, *p*=0.8219).

The agreement between the two testing methods was assessed using weighted Cohen’s Kappa. The results showed a κ of 0.6222 (95% confidence interval (CI) (0.217, 0.865), *p* < 0.001).

## 4 DISCUSSION

Our findings suggest that the IMMUNOWELL™ JCV test tends to classify a larger proportion of patients diagnosed with RRMS into higher risk categories for PML compared to the STRATIFY JCV™ DxSelect™ test. This difference could favor a shift to more conservative management strategies, potentially increasing the number of patients receiving intensive monitoring. The transition to the use of biosimilars as DMT for patients diagnosed with RRMS necessitates careful management to address potential differences in immunogenicity and regulatory pathways while still providing a comparable therapeutic alternative [20, 21]. Our results raise important considerations about the implementation of the findings of our study may raise significant questions about the use of IMMUNOWELL™ JCV for PML risk assessment in patients undergoing natalizumab therapy. IMMUNOWELL™ JCV test showed a consistent pattern of classifying patients into higher risk categories compared to the STRATIFY JCV™ DxSelect™ test, and this differential classification pattern could have important implications for clinical practice. The observed risk stratification differences between the two assays present a real-world clinical challenge, calling for a careful balance between risk mitigation and optimal therapeutic management.

Among the potential implications for clinical management protocols that might follow the differential risk stratification could be the implementation of more intensive monitoring, including greater frequency of magnetic resonance imaging, serial JCV antibody testing, and enhanced surveillance for PML-associated neurological manifestations. While this heightened surveillance may facilitate earlier detection of adverse events, it also introduces additional resource utilization considerations. The potential reclassification of patients into higher-risk categories could necessitate modifications to therapeutic strategies, including consideration of extended interval dosing or treatment discontinuation. This modified risk management approach, while potentially reducing PML risk, requires careful evaluation against the established efficacy of continuous natalizumab therapy in controlling disease activity. In other words, heightened caution following risk reclassification could compromise the efficacy of treatment for a subset of patients who could have safely continued therapy under previous risk assessment protocols. Such switches may expose patients to rebound inflammatory disease activity, even when transitioning to high-efficacy DMTs. Zhu *et al*. [22] reported an annualized relapse rate of 0.06 in patients switching from natalizumab to ocrelizumab, which, while low, remains higher than switches from platform therapies to ocrelizumab, such as those described by Foong *et al*. [23].

To address these concerns, future research should focus on longitudinal studies to validate these findings and assess the long-term clinical outcomes following risk classification according to the use of the IMMUNOWELL™ JCV test as well as on different cohorts (*i.e*., first DMT prescription, different kinds of therapeutic transition).

Additionally, there is a pressing need to develop effective strategies for communicating risk changes to patients and optimizing resource allocation in clinical settings. This may involve creating clear guidelines and educational materials to help patients make informed decisions about their treatment, as well as implementing risk-stratified care pathways and telemedicine solutions to manage the increased demand for efficient monitoring.

Our study has limitations. Firstly, the small sample size potentially limits the generalizability of results to broader populations. The absence of a priori power analysis may affect the reliability and generalizability of the results, highlighting the need for careful consideration of these factors in future research. The cohort selection itself, consisting exclusively of natalizumab-treated patients, also constitutes a methodological limitation. Furthermore, the presence of confounding variables and potential selection bias may have influenced the observed associations.

## CONCLUSION

To our knowledge, this is the first study to specifically compare these two tests for PML risk assessment, offering foundational insights that can guide future research and in-form clinical practice while maintaining a balanced perspec-tive on the integration of biosimilars into clinical practice.

Nonetheless, the comparative performance characteristics and risk stratification algorithms warrant further investigation through large-scale validation studies.

## Figures and Tables

**Table 1:** Characteristics of the enrolled cohort.

**Total Cohort (n=69)**	
Race, n(%) Caucasian	69 (100)
Age (years), mean ± SD	39 ± 10.3
Age at MS diagnosis (years), mean ± SD	28.2 ± 9.3
Sex, n (%)	Female: 53 (76.8) Male: 16 (23.2)
EDSS at NTZ prescription, median (IQR)	3.00 (2.0-4.0)
EDSS, median (IQR)	3.00 (2.5-4.0)
Patients with MRI activity in the last year, n(%)	0
Number of Relapses in the last two years	0.2 ± 0.5
Patients with >24 administrations, n(%)	47 (68.1)
Patients on EID regimen, n(%)	21 (30.4)
Patients previously exposed to immunosuppressive* drugs	4 (5.8)
Naïve to any DMT, n(%)	27 (39.1)
N. of NTZ Administrations, mean ± SD	46.7 ± 45.7
Time on NTZ (months), mean ± SD	68.7 ± 180.3

**Abbreviations:** SD, standard deviation; IQR, interquartile range; n, number; EDSS, Expanded Disability Status Scale; EID, extended interval dose; MS, multiple sclerosis; NTZ, natalizumab; MRI, magnetic resonance imaging; DMT, disease modifying therapy.

**Note:** *in our cohort: alemtuzumab, cladribine, azathioprine.

**Table 2:** Titer-based risk stratification: STRATIFY JCV™ DxSelect™ *vs*. IMMUNOWELL™ JCV antibody titers.

**Risk Level, n.**	**STRATIFY JCV™ DxSelect™**	**IMMUNOWELL™ JCV**	**Chi-square**	***p*-value**
High	3	5	37.8	0.0001
Intermediate	3	10	17.4	0.0002
Low	63	54	2.3	0.3041

**Note:** n. number of patients.

## Data Availability

The data and supportive information are available within the article.

## LIST OF ABBREVIATIONS

CI: Confidence Interval
DMT: Disease-modifying Therapy
JCV: J.C. Virus
PML: Progressive Multifocal Leukoencephalopathy
RRMS: Relapsing-remitting Multiple Sclerosis

## STANDARDS OF REPORTING

STROBE guidelines were followed.
